# Prevention of Vaginal SHIV Transmission in Macaques by a Coitally-Dependent Truvada Regimen

**DOI:** 10.1371/journal.pone.0050632

**Published:** 2012-12-04

**Authors:** Jessica Radzio, Wutyi Aung, Angela Holder, Amy Martin, Elizabeth Sweeney, James Mitchell, Shanon Bachman, Chou-Pong Pau, Walid Heneine, J. Gerardo García-Lerma

**Affiliations:** Laboratory Branch, Division of HIV/AIDS Prevention, National Center for HIV, Hepatitis, STD, and Prevention, Centers for Disease Control and Prevention, Atlanta, Georgia, United States of America; University of Pittsburgh, United States of America

## Abstract

**Background:**

Daily pre-exposure prophylaxis (PrEP) with Truvada (a combination of emtricitabine (FTC) and tenofovir (TFV) disoproxil fumarate (TDF)) is a novel HIV prevention strategy recently found to prevent HIV transmission in men who have sex with men and heterosexual couples. We previously showed that a coitally-dependent Truvada regimen protected macaques against rectal SHIV transmission. Here we examined FTC and tenofovir TFV exposure in vaginal tissues after oral dosing and assessed if peri-coital Truvada also protects macaques against vaginal SHIV infection.

**Methods:**

The pharmacokinetic profile of emtricitabine (FTC) and tenofovir (TFV) was evaluated at first dose. FTC and TFV levels were measured in blood plasma, rectal, and vaginal secretions. Intracellular concentrations of FTC-triphosphate (FTC-TP) and TFV-diphosphate (TFV-DP) were measured in PBMCs, rectal tissues, and vaginal tissues. Efficacy of Truvada in preventing vaginal SHIV infection was assessed using a repeat-exposure vaginal SHIV transmission model consisting of weekly exposures to low doses of SHIV162p3. Six pigtail macaques with normal menstrual cycles received Truvada 24 h before and 2 h after each weekly virus exposure and six received placebo. Infection was monitored by serology and PCR amplification of SHIV RNA and DNA.

**Results:**

As in humans, the concentration of FTC was higher than the concentration of TFV in vaginal secretions. Also as in humans, TFV levels in vaginal secretions were lower than in rectal secretions. Intracellular TFV-DP concentrations were also lower in vaginal tissues than in rectal tissues. Despite the low vaginal TFV exposure, all six treated macaques were protected from infection after 18 exposures or 4 full menstrual cycles. In contrast, all 6 control animals were infected.

**Conclusions:**

We modeled a peri-coital regimen with two doses of Truvada and showed that it fully protected macaques from repeated SHIV exposures. Our results open the possibility for simplified PrEP regimens to prevent vaginal HIV transmission in women.

## Introduction

Daily pre-exposure prophylaxis (PrEP) with oral Truvada, a combination of emtricitabine (FTC) and tenofovir (TFV) disoproxil fumarate (TDF), is a novel biomedical intervention recently found to prevent HIV transmission among high-risk populations. In the iPrEX trial among men who have sex with men and transgender women, the incidence of HIV-1 was reduced by 44% compared to placebo. Efficacy was increased to 73% among participants who reported adherence to the regimen, and by more than 90% in those that had detectable drug [1]. The CDC TDF-2 trial among HIV-negative heterosexual men and women showed an overall efficacy of 63% compared to placebo [2]. The Partners PrEP trial among serodiscordant couples found a strong HIV prevention effect with TDF (62% fewer infections) and Truvada (73% fewer infections) [3]. A fourth trial (FEM-PrEP) with Truvada among high-risk women was recently stopped due to futility because of low adherence [4]. Truvada is now approved by the Food and Drug Administration for PrEP to reduce the risk of sexually acquired HIV-1 in adults at high risk.

Simian/human immunodeficiency virus (SHIV) infection of macaques is a well-established model of HIV transmission that can be used to investigate the efficacy of oral and topical PrEP in preventing rectal and vaginal transmission [5], [6]. We have previously modeled the efficacy of daily oral FTC, TDF, and Truvada against rectal SHIV transmission in rhesus macaques and found that the combination of FTC and TDF at human equivalent doses provided a level of protection similar to that recently seen in the iPrEX trial among highly adherent participants [7]. We also found that intermittent Truvada regimens containing one dose given 1–7 days prior to exposure followed by a second dose 2 h after exposure, or two peri-coital doses administered within 24 h were as effective as daily PrEP [8]. While these studies suggested that intermittent PrEP might be an alternative to daily PrEP, all were focused on rectal transmission and cannot inform efficacy against vaginal transmission.

The efficacy of intermittent Truvada against vaginal transmission is difficult to predict from rectal efficacy data due to physiological and pharmacological differences between vaginal and rectal tissues. The multilayered squamous epithelium that covers the vagina differs from the single layer columnar epithelium of the rectum and has a unique immunological composition that may influence early establishment and dissemination of infection [9], [10], [11]. The vaginal mucosa also undergoes changes during the menstrual cycle that may influence susceptibility to infection and potentially affect antiretroviral drug penetration [12]. In humans, the concentrations of FTC, TFV, and their active intracellular metabolites (FTC-triphosphate (FTC-TP) and tenofovir-diphosphate (TFV-DP), respectively) differ among vaginal and rectal tissues [13]. In vaginal tissues, the cumulative exposure to TFV is 100-fold lower than in rectal tissues, while exposure to FTC in vaginal tissues is higher than exposure to TFV [13]. It is therefore important to explore if the low TFV penetration in vaginal tissues may potentially reduce the efficacy of intermittent PrEP with Truvada.

We determined if FTC and TFV concentrations achieved in vaginal tissues during a simplified 2-dose peri-coital Truvada regimen administered 24 h before and 2 h after vaginal SHIV exposures are sufficient to prevent SHIV transmission in pigtail macaques. We used pigtail and not rhesus macaques because pigtails have normal lunar menstrual cycles and changes in hormone levels similar to humans [14]. The pigtail macaque menstrual cycle averages 32.8 days and recapitulates potential fluctuations in susceptibility to SIV infection associated with the follicular and luteal phase of the menstrual cycle [12], [15], [16]. We first show that levels and ratios of FTC and TFV in vaginal tissues after oral administration of human-equivalent doses follow the same pattern as those seen in humans. We then demonstrate that these drug levels are sufficient to prevent vaginal SHIV infection in macaques, thus expanding our previous observations on rectal efficacy of coitally-dependent PrEP regimens with Truvada.

## Methods

### Ethics Statement

All the animal procedures performed in this study were approved by the Institutional CDC Animal Care and Use Committee (Protocols 2070GARMON and 2061GARMONC). Macaques were housed at the Centers for Disease Control and Prevention under the full care of CDC veterinarians in accordance with the standards incorporated in the Guide for the Care and Use of Laboratory Animals (National Research Council of the National Academies, 2010). SHIV infected macaques were humanely euthanized in accordance with the American Veterinary Medical Association Guidelines on Euthanasia, June 2007. All procedures were performed under anesthesia using ketamine, and all efforts were made to minimize suffering, improve housing conditions, and to provide enrichment opportunities (e.g., objects to manipulate in cage, varied food supplements, foraging and task-oriented feeding methods, interaction with caregivers and research staff).

### Drugs and Viruses

TDF and FTC (kindly provided by Gilead Sciences) were prepared as previously described and were given orally by gavage based on body weight as a single solution containing 20 mg/kg of FTC and 22 mg/kg of TDF [8]. Macaques were anesthetized using standard doses of ketamine hydrochloride. The SHIV_162P3_ virus challenge stock was obtained from the NIH AIDS Research repository and expanded in pigtail macaque peripheral blood mononuclear cells (PBMCs) prior to this study.

### Measurement of FTC and TFV Levels in Plasma, Vaginal Secretions, and Rectal Secretions

FTC and TFV concentrations were measured in 6 pigtails macaques after a single oral dose of Truvada. Rectal and vaginal secretions were collected in sponges (Weck-Cel Surgical Spear, Medtronic Ophthalmic, Jacksonville, FL) using an established protocol [8], [17]. Concentrations of FTC and TFV were measured by HPLC-MS/MS as recently described [18]. The method provides simultaneous measurement of FTC and TFV with a limit of quantification of 5 ng/mL [18]. Final drug concentrations are expressed as ng per ml of plasma, rectal, or vaginal secretion.

### Intracellular FTC-TP and TFV-DP Levels in PBMCs and Tissues

Intracellular FTC-TP and TFV-DP concentrations were measured in two groups of macaques. The first group (n = 6) received a single oral dose of Truvada followed by blood collection and FTC-TP and TFV-DP measurements in PBMCs. Red blood cells were lysed to minimize interferences in intracellular TFV-DP determinations [19]. A second group of animals (n = 6) received a single oral dose of Truvada followed by necropsy and blood and tissue collection at 24 h (3 macaques) or 3 days (3 macaques). Drug levels were measured in cell suspensions, and/or whole biopsies obtained from vaginal, rectal, and lymphoid tissues (axillary, mesenteric, and inguinal lymph nodes). Cell suspensions were prepared from vaginal and rectal tissues using an enzyme cocktail containing collagenase type II, elastase, hyaluronidase, and DNase I, and contain mostly mononuclear and epithelial cells [8]. FTC-TP and TFV-DP levels were measured as described previously [20].

### Repeat Exposure Vaginal SHIV Transmission Model

The efficacy of Truvada in preventing vaginal transmission was evaluated using an established pigtail macaque model consisting of repeated vaginal exposures to a low dose (50 TCID_50_) of an R5-tropic SHIV_162P3_ isolate [6], [21]. Vaginal exposures were performed once a week for up to 18 weeks by non-traumatic inoculation of 1 mL of SHIV_162P3_ into the vaginal vault via a sterile gastric feeding tube of adjusted length; virus exposures were stopped when a macaque became SHIV RNA positive [6]. Anesthetized macaques remained recumbent for at least 15 min after each intra-vaginal inoculation. Six of the pigtail macaques received a Truvada regimen consisting of an oral dose given 24 h before each virus exposure followed by a second dose 2 h post exposure. Six macaques received PBS and were used as placebo controls. All experiments were done under highly controlled conditions by the same personnel, and using fresh aliquots of the same virus stock stored in liquid nitrogen.

### Infection Monitoring by Molecular and Serologic Testing

SHIV RNA was quantified in plasma, rectal, and vaginal secretions. Rectal and vaginal secretions were collected in sponges only after confirmed infection. Briefly, 2 dry sponges were inserted 5 cm into the rectum or vaginal cavity of recumbent macaques and maintained during 5 minutes. Sponges were then added to 1 mL of PBS and incubated at room temperature during 15 minutes. SHIV RNA from plasma (1 mL or 100 uL) and secretions (1 mL) was quantified by RT-PCR [22]. The amount of virus in secretions was expressed as RNA copies per sponge (each containing an average of 30 uL of secretion) and includes a correction to account for plasma-derived viruses originating from blood contamination. The correction has been described elsewhere [23] and takes into consideration hemoglobin levels in secretions (measured using Multistix 7 Reagent Strips, Mountainside Medical Equipment, Marcy, NY), individual macaque hematocrit values, hemoglobin concentrations, and plasma SHIV RNA levels.

Proviral DNA was monitored in PBMCs as previously described [8]. Virus-specific serologic responses were measured using a synthetic-peptide EIA (BioRad, Genetic Systems HIV-1/HIV-2, Redmond, WA) assay. Animals were considered protected from systemic SHIV infection if they remained seronegative and negative for SHIV plasma RNA and SHIV DNA in PBMCs during the 18 weeks of PrEP and the following 18 weeks of drug washout.

### Determination of Progesterone Levels in Plasma

Progesterone levels in plasma were quantified using a progesterone EIA Kit (Cayman Chemical, Ann Aarbor, MI). Samples were tested neat or pre-diluted 1∶10 in the supplied EIA buffer and processed as recommended by the manufacturer.

## Results

### Pharmacokinetic Profile of TFV and TFV in Vaginal Secretions from Pigtail Macaques

**Figure 1:**
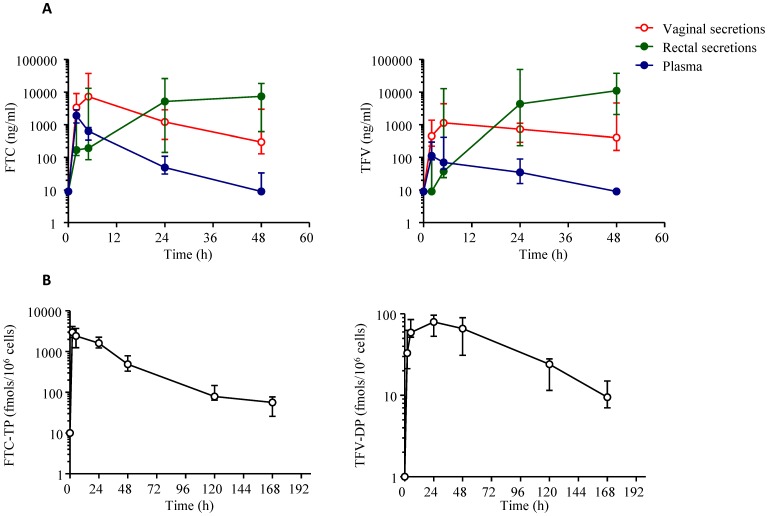
Pharmacokinetic profile of FTC and TDF in pigtail macaques. A: Concentrations of FTC and TFV in plasma, rectal, and vaginal secretions after a single oral dose of FTC and TDF. Secretions were collected in 6 macaques using sponges at the indicated time points. Median concentrations and interquartile ranges (vertical lines) are shown. B: Intracellular FTC-TP and TFV-DP concentrations in pigtail macaque PBMCs. Six pigtail macaques received a single oral dose of FTC and TDF by gavage followed by collection of blood at the indicated time. Median concentrations and interquartile ranges (vertical lines) for FTC-TP (1) and TFV-DP (2) are shown.

We have shown that an oral dose of 20 mg/kg TDF and 22 mg/kg FTC given to rhesus macaques resulted in systemic drug exposures that were within the range of those seen in humans [8]. We explored if the same drug doses administered to pigtail macaques result in similar drug levels. Figure 1 shows that, at these doses, peak TFV and FTC levels (median = 113 and 1,920 ng/mL, respectively), T_max_ (median = 2 h), and area under the curve values over 24 h (AUC_0–24 h_, median = 1,994 and 12,273 ng/h•mL) were similar to those in humans [8], [24]. In PBMCs, he estimated intracellular half-life of TFV-DP was 58 h(range, 39–66 h) and the intracellular half-life of FTC-TP was 29 h (range, 22–32 h) (Figure 1).

We next determined the levels of FTC and TFV in vaginal secretions and compared it with that seen in rectal secretions (Figure 1). FTC and TFV concentrations in vaginal secretions peaked at 5 h (median = 7,230 and 1,135 ng/mL, respectively) and remained higher than in plasma during 24 h–48 h. In rectal secretions, FTC and TFV levels were low during the first 5 h and increased substantially between 24 h and 48 h. The median AUC_0–48 h_ value for FTC in vaginal and rectal secretions were 194,447 and 427,077 ng**•**h/mL, respectively. Similarly, the median AUC_0–48 h_ value for TFV in vaginal and rectal secretions were 51,235 and 680,336 ng**•**h/mL, respectively. We calculated AUC_0–48 h_ ratios as a measure of relative drug exposure in vaginal and rectal tissues. For FTC, the median vaginal:rectal AUC_0–48 h_ ratio was 0.46 (range = 0.1–9.8). For TFV, the median vaginal:rectal AUC_0–48 h_ ratios was 0.08 (0.02–0.7) demonstrating that as in humans, TFV exposure in vaginal tissues from pigtail macaques is substantially lower than in rectal tissues [13]. We also evaluated the ratios between FTC and TFV concentrations. In vaginal secretions, the median FTC:TFV AUC_0–48 h_ ratio was 3.8 (range = 0.9–12.8) demonstrating that, also as in humans, FTC exposure in vaginal tissues is higher than TFV exposure [13]. In rectal secretions, the median FTC:TFV AUC_0–48 h_ ratio was 0.63 (range = 0.5–1.2). Overall, these findings suggest that 20 mg/kg TDF and 22 mg/kg FTC administered orally to pigtail macaques recapitulate the degree of systemic and vaginal FTC and TFV exposure seen in humans.

### Intracellular FTC-TP and TFV-DP Levels in Vaginal Tissues

We next measured TFV-DP and FTC-TP levels in vaginal tissues by giving a single oral dose of Truvada to 6 macaques followed by necropsies to collect biopsies and tissues after 24 h (3 animals) or 3 days (3 animals). TFV-DP and FTC-TP concentrations were also measured in rectal tissues and biopsies, and in axillary, mesenteric, and inguinal lymph nodes. Table 1 shows that intracellular TFV-DP concentrations in vaginal cells or biopsies at 24 h (24 fmols/10^6^ cells or 4 fmols/mg tissue) were 25-fold (on a per cell basis) or 40-fold (on a per mg of tissue basis) lower than those seen in rectal cells or biopsies (634 fmols/10^6^ cells or 166 fmols/mg tissue). Vaginal TFV-DP levels were also lower than rectal concentrations at 3 days albeit to a lesser degree. Table 1 show that at 3 days, TFV-DP levels in vaginal cells (18 fmols/10^6^ cells) or biopsies (2 fmols/mg tissue) were 6-fold and 13-fold lower than those seen in rectal cells or biopsies (110 fmols/10^6^ cells or 27 fmols/mg tissue, respectively). Overall, these data confirm that, as in humans, TFV exposure in vaginal tissues from pigtail macaques is lower than in rectal tissues.

**Table 1:** Intracellular TFV-DP and FTC-TP concentrations at 24 h and 3 days in vaginal, rectal, and lymphoid tissue after a single oral dose.

	TFV-DP				FTC-TP			
	fmols/10^6^ cells		fmols/mg tissue		fmols/10^6^ cells		fmols/mg tissue	
	24 h	3 days	24 h	3 days	24 h	3 days	24 h	3 days
Vaginal	24 (22–39)	18 (6–32)	4 (4–5)	2 (2–9)	122 (91–138)	80 (25–183)	151 (145–179)	10 (6–22)
Rectal	634 (11–783)	110 (51–336)	166 (5–697)	27 (18–44)	117 (44–125)	56 (19–169)	72 (24–118)	9 (5–21)
Lymphoid (mesenteric, axillary, inguinal)	21.5 (14–39)	25 (16–57)	6 (3–17)	7 (3–11)	622 (437–784)	221 (62–345)	76 (25–115)	13 (5–55)
Relative penetrationVT:RT	0.04	0.16	0.02	0.07	1.04	1.42	2.10	1.11

### Efficacy of Intermittent Truvada in Preventing Vaginal SHIV Transmission

We then investigated if the concentrations of TFV and FTC achieved in vaginal tissues could prevent SHIV infection. Macaques were exposed to SHIV_162P3_ once a week for up to 18 weeks. Because the average menstrual cycle in these macaques was 4.4 weeks or 30 days (see below), the 18 weeks of virus exposures are equivalent to at least 4 full menstrual cycles. Six of the 12 macaques received two weekly doses of Truvada and 6 received placebo. The average age (14.5 and 12.5 years), weight (8.5 and 7.91) and peak progesterone levels (1,996 and 1,835 ng/mL) measured prior to virus challenges did not differ significantly between the treated and placebo group (*p*>0.5 for each comparison, Mann-Whitney two-tailed *t*-test).

**Figure 2:**
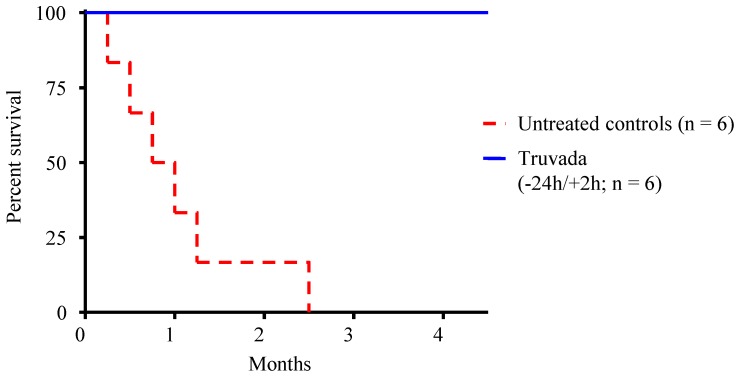
Complete protection against vaginal SHIV transmission by intermittent PrEP with Truvada. Survival curves represent the cumulative percentage of uninfected macaques as a function of the number of months in the study period (4 challenges per month). Control macaques become infected after a median of 3.5 exposures or about 1 menstrual cycle. Virus challenges in the macaques receiving Truvada were stopped after 18 SHIV_SF162P3_ exposures or about 4.5 menstrual cycles. Protected animals remained seronegative and RNA/DNA negative during a follow up period of 18 weeks.

**Figure 3:**
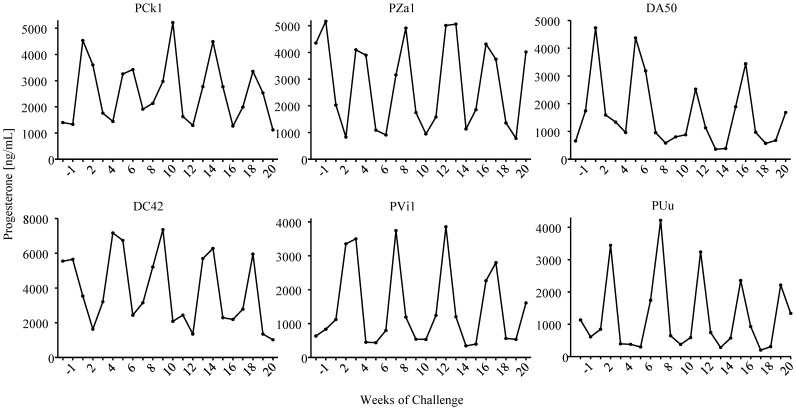
Plasma progesterone levels in macaques receiving oral Truvada. Animals remained protected from infection for an average of 4 complete menstrual cycles as indicated by the fluctuations in progesterone levels overtime.

**Figure 4:**
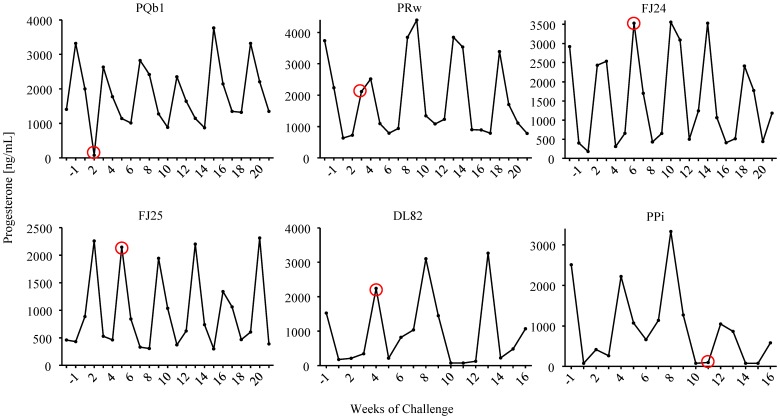
Plasma progesterone levels in placebo control macaques. The red circles indicate the first detection of viral RNA in plasma. Exposures were stopped when a macaque became SHIV RNA positive in plasma.

All 6 animals that received two weekly doses of Truvada remained seronegative and viral RNA and DNA negative during the 18 virus challenges and a follow-up period of 18 additional weeks, demonstrating that vaginal and systemic FTC and TFV levels achieved during this PrEP regimen were sufficient to prevent infection (Figure 2). Figure 3 shows that all macaques were protected during at least 4 full menstrual cycles. In contrast, all 6 animals receiving placebo were infected after a median of 1 menstrual cycle (range 1–3) or 3.5 exposures; animals were infected at exposures 1, 2, 3, 4, 5, or 10 (Figure 2). Figure 4 illustrates the time of first RNA detection during the menstrual cycle in these six untreated control macaques.

### Plasma Viremia and Vaginal and Rectal Virus Shedding in Infected Macaques

**Figure 5:**
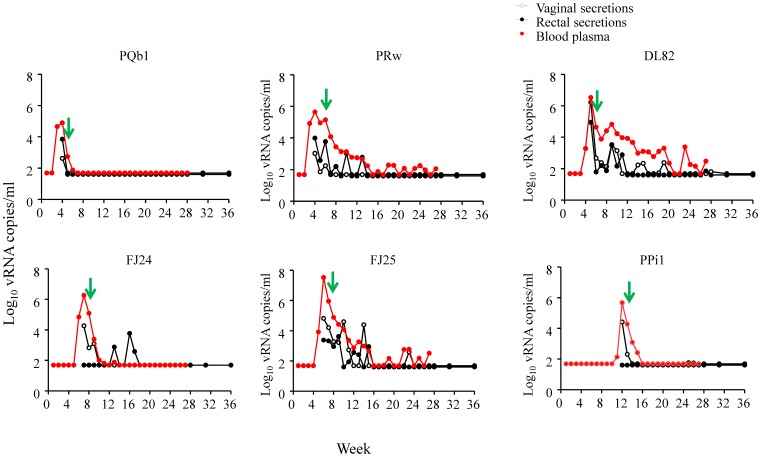
Plasma viremia, rectal and vaginal shedding, and seroconversion in infected placebo controls. SHIV RNA levels in plasma (red circles), vaginal secretions (open circle), and rectal secretions (closed cycles) were measured weekly for up to 38 weeks after the first virus challenge. Exposures were stopped when a macaque became SHIV RNA positive in plasma. The first detectable antibody response is shown as a green arrow.

Figure 5 shows viral dynamics in plasma, vaginal, and rectal secretions from the six placebo controls. Average peak virus load in plasma was 6.7×10^6^ RNA copies/mL (range = 1.9×10^6^ and 3.4×10^7^). The overall kinetics of virus shedding in vaginal and rectal secretions were similar to those seen in plasma (Figure 5). RNA levels in vaginal or rectal secretions correlated with plasma levels (Pearson correlation coefficient, *r* = 0.745 (95% CI = 0.658–0.813) for vaginal/plasma, and 0.545 (95% CI = 0.405–0.660) for rectal/plasma, two-tailed *p*<0.0001 for both comparisons). RNA levels in rectal and vaginal secretions also correlated (*r* = 0.292 (95% CI = 0.118–0.448), two-tailed *p* = 0.0013).

## Discussion

We demonstrate that oral Truvada administered peri-coitally protects pigtail macaques from vaginal SHIV transmission. We used pigtail macaques because they have regular lunar menstrual cycles and plasma sex hormone changes similar to humans [25]. We show that a simple Truvada regimen consisting of only 2 doses (one 24 h before and another 2 h after exposure) resulted in complete protection from infection. These findings expand our previous observations in rhesus macaques where the same Truvada regimen was also highly protective against rectal virus transmission [8]. The high prophylactic efficacy demonstrated with a coitally-dependent Truvada regimen against both vaginal and rectal transmission in macaques suggest that this PrEP modality might be an alternative to daily dosing, and informs clinical evaluation of exposure-driven intermittent PrEP regimens for preventing sexual HIV transmission.

Our analysis of FTC and TDF PK profile was key to ensure that we are modeling doses of Truvada similar to those used in humans. The doses of FTC and TDF were adjusted for the weight and size of macaques since smaller mammals usually eliminate drugs faster than larger mammals [26]. At these doses, C_max_, T_max_ and AUC values for TFV and FTC in plasma were within the range seen in humans [24]. Also as in humans, the half-life of TFV-DP and FTC-TP was long in PBMCs (about 60 and 24 h respectively). However, several limitations precluded a better comparison with available human data. First, our FTC-TP and TFV-DP PK analysis in PBMCs and rectal tissues was done at first dose and did not allow a direct comparison with human data which is only available at steady state [13], [27], [28], [29]. It will be important to compare our absolute drug levels and drug half-life once first dose kinetics become available in humans. Second, a direct comparison of drug levels in vaginal secretions between macaques and humans was not possible due to differences in collected methods (wicks compared to direct aspiration), sampling methodologies, and testing procedures. However, we found that, as in humans, FTC and TFV peaked later in vaginal secretions than in plasma and remained higher than plasma levels over a 48 h period [24]. Also as in humans, the concentration of FTC in vaginal secretions was higher than the concentration of TFV, and both TFV and TFV-DP levels in vaginal tissues were substantially lower than in rectal tissues [13]. Interestingly, the differences in TFV-DP levels between vaginal and rectal tissues appeared to wane overtime, suggesting that TFV-DP half-life in rectal tissues might be reduced compared to vaginal tissues. If confirmed in larger number of animals and in humans, this observation suggests that rectal efficacy of intermittent PrEP based on fixed drug dosing Truvada regimens (e.g., weekly), may not necessarily benefit from higher rectal TFV-DP levels.

The differential penetration of FTC and TFV in the rectal and vaginal compartments raises questions regarding the relative contribution of FTC and TFV to the efficacy of daily Truvada seen in different risk populations [1], [2], [3], [13]. The finding that daily TDF alone prevented HIV transmission in highly adherent women demonstrates that the antiviral activity resulting from daily dosing is sufficient to prevent vaginal transmission [3]. Given the low vaginal TFV exposure in women, it is possible that both the systemic and vaginal drug activity contribute to protection. Additional studies in macaques using for instance vaginal gels that result in tissue TFV-DP levels similar to those achieved after oral dosing may help to understand the relative contribution of local vs systemic TFV levels to the observed protection. It will also be interesting to determine in our monkey model if intermittent dosing with TDF only is equally protective to Truvada. Although the efficacy of daily TDF in preventing rectal HIV transmission in humans is not known, macaque studies with TDF or another TFV prodrug predict lower efficacy against rectal transmission and suggest that, for rectal efficacy, Truvada may be required [22], [30].

Our study is subjected to several limitations. First, all virus challenges were non-traumatic and done in the absence of semen or semen-derived factors that may enhance susceptibility to HIV infection or other cofactors that may increase HIV transmission risk, such as sexually transmitted infections. Second, we did not model fixed-dose intermittent PrEP regimens containing for instance 2 weekly doses of Truvada followed by a booster post-exposure dose as in the ADAPT study [31]. Although fixed-dosing PrEP modalities were highly efficacious against rectal transmission in macaques, rectal efficacy results cannot be extrapolated to vaginal efficacy given the differences in drug penetration between vaginal and rectal tissues [8]. Third, we did not measure drug levels in the interior iliac lymph nodes. This is important since these nodes drain both the genital and rectal tract and are believed to be an important site of early virus replication [32].

Vaginal transmissibility studies in macaques have conventionally used the injectable contraceptive Depo-Provera (DMPA) to maximize infection of animals [33], [34], [35]. Our experimental design did not include DMPA because we wanted to fully recapitulate the variabilities in susceptibility to infection associated with the menstrual cycle as well as evaluate protection over repeated virus exposures [16]. However, the use of DMPA may be useful to model the potential impact of injectable contraceptives on the efficacy of PrEP with Truvada. This analysis will be important since recent studies have suggested an increased risk of HIV acquisition associated with the use of DMPA, and PrEP clinical trials with different patterns of hormonal contraceptive use have shown contradictory results [2], [3], [4], [36]. The development of a biologically relevant macaque model to determine the effect of DMPA on PrEP efficacy will first require careful selection of DMPA doses that mimic human exposure and biological effects [37]. It will also be important to explore if other factors such as sexually transmitted infections might reduce efficacy among highly adherent PrEP users.

Our analysis of virus levels in vaginal and rectal tissues showed high levels of virus shedding accompanying peak plasma viremia after correcting for blood contamination in secretions. We found virus levels as high as 1×10^6^ copies per wick despite the low volume of rectal and vaginal secretion contained in a single wick (30–35 uL). These results are consistent with the increased HIV transmissibility seen during the acute phase of HIV infection [38].

The acceptability of intermittent PrEP in different high-risk groups as well as the capacity of individuals to anticipate sexual activity and consistently adhere to intermittent dosing is not known [39]. Reported adherence to intermittent PrEP was recently found to be lower than daily dosing among MSM and female sex workers [40]. However, only a small number of men and women participated in this study and measurements of adherence to coitally dependent regimens using medication event monitoring systems or short message service was particularly challenging [40]. An ongoing, larger Phase II trial among MSM and women who have sex with men is currently evaluating adherence to daily and intermittent Truvada dosing, and is also exploring intracellular drug levels at given rates of adherence during direct observed therapy [31]. This study will better define behavioral aspects associated with intermittent dosing, coverage of sex events with pre-and post-exposure dosing, and if intermittent PrEP may achieve intracellular drug levels that cross the pharmacological barrier associated with prevention of HIV acquisition. The later aspect is particularly important since the same level of adherence might have a different impact on rectal and vaginal efficacy given the differences in FTC and TFV penetration among rectal and vaginal tissues.

In summary, we used a pigtail macaque model to evaluate a before/after exposure prophylactic modality that included 2 doses of Truvada within 24 h, and showed a high effectiveness in preventing vaginal SHIV transmission. These findings suggest that Truvada might prevent vaginal transmission in humans if taken peri-coitally. Less frequent drug administration would reduce cost and might decrease drug toxicities by reducing unnecessary drug exposure and frequency of mild side effects. It will be important to determine the acceptability of coitally-dependent PrEP based on ongoing studies among different high-risk populations, and if individuals will be able to anticipate sexual activity and consistently adhere [39]. Specific populations such as serodiscordant couples planning to conceive a child might particularly benefit from coitally-dependent PrEP.
